# Deficits in Cognitive–Motor Control of the Ipsilesional Upper Limb in Subacute Stroke Assessed Using a Robotic Exoskeleton: A Longitudinal Study

**DOI:** 10.3390/brainsci16060595

**Published:** 2026-05-30

**Authors:** Emmanuel Segnon Sogbossi, Léandre Gagné-Pelletier, Catherine Mercier

**Affiliations:** 1 School of Rehabilitation Sciences, Faculty of Medicine, Université Laval, Quebec City, QC G1V 0A6, Canada; segnon-emmanuel-augustin.sogbossi.1@ulaval.ca (E.S.S.); leandre.gagne-pelletier.1@ulaval.ca (L.G.-P.); 2 Centre for Interdisciplinary Research in Rehabilitation and Social Integration (Cirris), CIUSSS de la Capitale-Nationale, Quebec City, QC G1M 2S8, Canada

**Keywords:** cognitive control, motor control, visuomotor, stroke, rehabilitation

## Abstract

**Highlights:**

**What are the main findings?**

- Stroke survivors exhibit specific deficits in cognitive–motor control during visuomotor tasks early after stroke, which persist into the chronic stage.
- Lesions in the right hemisphere are linked to more severe impairments, suggesting a possible hemispheric specialization for cognitive–motor control in these tasks.

**What are the implications of the main findings?**

- Cognitive–motor control should be assessed early after a stroke.
- Rehabilitation interventions should consider the side of the lesion to target specific deficits associated with hemispheric lateralization.

**Abstract:**

**Background/Objectives**: This study longitudinally assessed cognitive–motor control deficits in the ipsilesional upper limb following stroke and, secondarily, examined the effect of lesion laterality on these deficits. **Methods**: Forty-one participants (mean [SD] age: 64.6 [14.4] years; 24 with right-hemisphere lesion; 38 right-handed) were assessed using the KINARM Exoskeleton Lab at approximately 4 weeks (T1), 10 weeks (T2), and 29 weeks (T3) post-stroke. They completed the Visually Guided Reaching (VGR) and Reverse Visually Guided Reaching (RVGR; where the cursor moved in the opposite direction to the subject’s hand movement) tasks with their ipsilesional limb to assess motor control and cognitive–motor control, respectively. Global Task-scores and Z-scores for specific variables derived from normative data were used to determine the occurrence of deficits within each task. Linear mixed-effects models examined time and lesion-side effects. **Results**: About 88% and 56% of participants were impaired on the RVGR global Task-score, at T1 and T3, respectively. In contrast, only 12% and 9% of participants were impaired on the VGR Task-score, at T1 and T3, respectively. Performance on the RVGR task improved over time. Interestingly, deficits were significantly more severe for right-hemisphere lesions on several variables, except for the feedforward variables. Performance on the VGR task remained unchanged with no lesion-side effect. **Conclusions**: Stroke survivors exhibited significant impairments in cognitive–motor control of the ipsilesional upper limb, independent of pure motor deficits, persisting into the chronic stage. Right-hemisphere lesions were associated with greater impairments, indicating a potential hemispheric specialization for such cognitive–motor control task.

## 1. Introduction

Stroke is a leading cause of long-term disability worldwide. According to the World Stroke Organization, the global burden of stroke increased substantially between 1990 and 2021, with an estimated 70% rise in incident strokes and a 44% increase in stroke-related deaths, largely driven by population growth and aging [1].

Stroke commonly results in hemiparesis, a sensorimotor deficit affecting predominantly one side of the body contralateral to the lesioned hemisphere. Within the first week following stroke onset, up to 80% of individuals exhibit motor impairments, with approximately 50% continuing to experience deficits at 6 months post-stroke [2,3,4]. Importantly, impairments are not limited to the contralesional side. The ipsilesional upper limb can also be affected, with up to 50% of stroke survivors demonstrating motor deficits within the first month, and approximately 15% showing persistent deficits at 6 months post-stroke [4,5]. These sensorimotor impairments can significantly compromise the ability to perform activities of daily living [6,7].

Beyond motor deficits, cognitive impairments are also prevalent after stroke and may persist for years [8]. These deficits commonly involve attention, working memory, and inhibitory control [8,9]. Increasing evidence highlights a strong interplay between cognitive and motor functions, such that elevated cognitive load can negatively impact motor performance in individuals with stroke [10,11,12]. This cognitive–motor interaction is critical for everyday functioning and underscores the need for sensitive and efficient tools to assess cognitive–motor control.

To date, a few studies have investigated upper limb-related cognitive–motor interference using dual-task paradigms, in which individuals perform separate cognitive and motor tasks simultaneously [12,13,14,15]. Other studies have explored associations between cognitive impairments, measured using standardized clinical assessments, and motor deficits [11,16,17]. While these approaches have contributed significantly to our understanding of cognitive–motor interactions, the impact of stroke on the online cognitive control of movement planning and execution remains relatively underexplored [5,18]. Online cognitive control of movements refers to the capacity to integrate task-relevant cognitive rules into the real-time initiation and execution of motor actions. This dynamic coupling between cognitive control processes and motor execution is essential for goal-directed behavior in everyday contexts, such as mirror-guided actions (e.g., combing one’s hair or shaving).

Robotic and computerized assessment tools offer a promising avenue to address this gap [5,18]. The Reverse Visually Guided Reaching (RVGR) task, implemented using the KINARM robotic exoskeleton, enables precise and quantitative assessment of cognitive–motor control. This task evaluates processes such as visuomotor transformation, inhibitory control, and attention during goal-directed movements [5,19,20]. Deficits on the RVGR task have been observed in individuals with subacute or chronic stroke [5,18] and other neurological populations including transient ischemic attack [21], epilepsy [19], and Parkinson’s disease [20]. However, longitudinal data on the RVGR task following stroke remain limited. To our knowledge, only one recent study has longitudinally followed a small cohort of subacute stroke participants (*n* = 14), reporting changes in overall performance on the RVGR task but not in specific metrics indexing distinct components of movement initiation and execution [5]. These limitations underscore the need for larger studies to confirm and extend these findings.

Moreover, studies on hemispheric dominance suggest that visuospatial attentional abilities are bilaterally represented but are more strongly lateralized to the right hemisphere [22,23,24]. Consequently, lesions in the right hemisphere are more likely to result in severe visuospatial attentional neglect affecting both visual fields, although the contralateral (left) visual space is typically more impaired [22,23,24]. This suggests that the right hemisphere may play a significant role in RVGR task performance, highlighting the importance of considering the effect of lesion laterality on RVGR outcomes.

The primary aim of this study was to longitudinally assess cognitive–motor control of the ipsilesional upper limb from the subacute phase (within 1 month post-stroke) to the chronic phase (6 months post-stroke). Focusing on the ipsilesional limb minimizes the confounding effects of paresis typically observed in the contralesional upper limb, allowing for a more precise evaluation of cognitive–motor control. The secondary aim was to examine the association between the lesioned hemisphere and the performance on cognitive–motor control tasks. We hypothesized that performance would be impaired in most individuals during the subacute phase but would improve over time. Furthermore, based on previous findings [5,10,18], we anticipated that individuals with right-hemisphere lesions would exhibit a lower performance compared to those with left-hemisphere lesions.

## 2. Materials and Methods

### 2.1. Study Design and Participants

This was a longitudinal cohort study, approved by the hospital ethic board (CIUSSS-CN, #2022-2550), and all participants provided written informed consent.

Patients admitted to the stroke rehabilitation unit of the Quebec Physical Rehabilitation Institute (CIUSSS-CN, QC, Canada) were systematically screened for eligibility between May 2022 and December 2024. Participants were approached to participate if they met the following inclusion criteria: (1) diagnosis of ischemic or hemorrhagic stroke confirmed by a neurologist; (2) less than 6 weeks post-stroke at the time of consent; and (3) hemiparesis affecting the upper limb. Exclusion criteria were: (1) inability to follow verbal commands; (2) history of prior symptomatic stroke or other neurological disorders; and (3) pre-existing musculoskeletal condition affecting the upper limb or stroke-related upper limb pain (typically in the shoulder) impeding the performance of robotic tasks.

Clinical and demographic information was extracted from patients’ medical records to characterize the sample. Cognitive status and visuospatial neglect were assessed using the Montreal Cognitive Assessment (MoCA) [25] and Bells test [26] administered by the unit’s neuropsychologist upon admission. The presence of visuospatial neglect was concluded following normative data reported by Mancuso et al. [26].

All participants underwent at least six weeks of intensive inpatient rehabilitation. This included four 1 h occupational therapy sessions and four 1 h physical therapy sessions. Additional therapies (e.g., task-specific group programs like GRASP or gait and balance training, and speech therapy) were provided as needed based on individual requirements.

### 2.2. Assessment Procedure

Eligible participants were assessed using a comprehensive battery of assessments at three time points: T1—upon admission to the stroke rehabilitation unit (~4 weeks post-stroke, range: 2–6 weeks); T2—six week later, prior to discharge (~10 weeks post-stroke, range 7–12 weeks); and T3—at follow up (~29 weeks post-stroke, range: 25–39 weeks).

The present study focused on two robotic-based evaluations of the ipsilesional upper limb. These included the Visually Guided Reaching (VGR) task, which assesses motor control, and the Reverse Visually Guided Reaching (RVGR) task, which evaluates cognitive–motor control [5,27].

During each session, participants were first positioned in the KINARM device (KINARM, Kingston, ON, Canada) and completed the VGR task. This was followed by additional experimental tasks, lasting approximately 25 min, before participants performed the RVGR task. The robot was used solely as an assessment tool.

#### 2.2.1. Robotic-Based Assessment

The KINARM Exoskeleton Lab (KINARM, Kingston, ON, Canada) has been extensively described in the literature and is widely used across a range of clinical populations [4,19,20,27]. The device provides gravitational support to the upper limb segments (arms, forearms, and hands), enabling movements of the shoulder and elbow in the horizontal plane.

**Figure 1:**
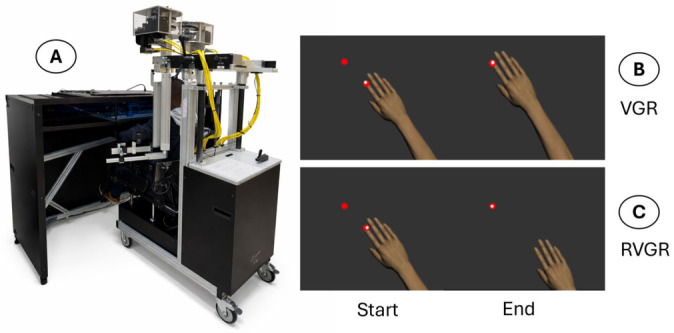
Illustrations of the KINARM robot exoskeleton (**A**), the Visually Guided Reaching (VGR, (**B**)) task and the Reverse Visually Guided Reaching (RVGR, (**C**)) task. In the VGR and RVGR tasks, targets are displayed in red, and the index finger is represented by a white dot.

Participants were seated in the adjustable KINARM chair (KINARM, Kingston, ON, Canada), with their shoulders abducted to approximately 85°. Participants’ foreheads were positioned against the virtual reality display, while both upper limbs were supported by the exoskeleton, preventing any trunk compensation during the reaching movement. A two-dimensional virtual reality interface was used to display the experimental tasks and provide real-time visual feedback of index finger position, represented as a white cursor. Direct visual feedback of the participant’s arms was occluded throughout testing (Figure 1).

*The Visually Guided Reaching (VGR)*: Participants were instructed to move as quickly and accurately as possible to one of four peripheral targets, presented sequentially, using a cursor (a white dot representing the position of the participant’s index finger; see Figure 1). The targets were arranged radially at 90° intervals and positioned 10 cm from a central starting location. For each trial, participants initiated movement from the central target, reached toward a peripheral target, and then returned to the center before proceeding to the next target. Peripheral targets appeared in a pseudo-random sequence with an inter-target interval ranging from 750 to 1250 ms, resulting in a total of 24 reaching movements [4,27]. All movements were performed in the workspace corresponding to the ipsilesional upper limb. Participants were instructed as follows: “*In this task, a white dot is at your index finger. Your objective is to move the white dot as quickly and accurately as you can from one red target to another red target. Hold that position until the next red target appears.*”

*The Reverse Visually Guided Reaching (RVGR)* task is structurally similar to the VGR task, with the critical distinction being that the cursor moves in the opposite direction to the participant’s hand movement (i.e., a 180° visuomotor rotation of cursor movement; see Figure 1). This reversed visuomotor mapping imposes increased cognitive demands, requiring participants to inhibit the automatic response of moving toward the visual stimulus and instead apply a rule-based transformation to guide their movement. As such, the task engages cognitive processes including inhibitory control, visuomotor transformation, and attention [5]. Participants received standardized task instructions as follows: “*In this task, a white dot is at your index finger. Your objective is to move the white dot as quickly and accurately as you can from one red target to another red target. Hold that position until the next red target appears. After the first target has been reached, you will notice that the white dot moves in the opposite direction of your hand. You still move the white dot to the red target*.”

#### 2.2.2. Performance Metrics

The csv files obtained from the KINARM for each task provided the Z-score for each evaluated task-specific variable and a composite score (*Task-score*). The Z-scores of the task-specific variables were obtained by transforming the raw score using a model of healthy controls accounting for age, sex, and handedness (see https://kinarm.com/kinarm-products/kinarm-standard-tests). 

In addition to the Task-score, 8 task-specific variables were obtained for both the VGR and RVGR tasks: Postural Speed, Reaction Time, Initial Direction Angle, Initial Distance Ratio, Speed Maxima Count, Min-Max Speed, Movement Time and Path Length Ratio.

Two additional task-specific variables were obtained for the RVGR task to specifically capture cognitive–motor control: Direction Errors and Correction Time.

Detailed definitions of each task-specific variable are provided in Table 1 and in the KINARM Standard Test Manual for calculation methods (see https://kinarm.com/kinarm-products/kinarm-standard-tests). Table 1 also identifies the more general category with which each task-specific variable is associated, highlighting the fact that these variables reflect different components of movement, such as feedforward vs. feedback component.

### 2.3. Data Analysis

Descriptive statistics were used to characterize the sample, with means and standard deviations reported for continuous variables and frequencies for categorical variables.

For the robotic tasks, impairments were estimated based on the overall Task-score and on the Z-scores for task-specific variables, with performance considered impaired when falling outside 95% confidence interval of controls. Task-scores are positive values with 0 denoting best performance and increasing values representing worse performance. Task-score values greater than 1.96 (i.e., falling outside of the 95% confidence interval of healthy controls) were considered indicative of impairment (see https://kinarm.com/kinarm-products/kinarm-standard-tests). For task-specific Z-score variables, values are signed and therefore values below or above ±1.65 (still corresponding to values outside of the 95% confidence interval of healthy controls) were considered impaired, depending on the direction of the variable (see https://kinarm.com/kinarm-products/kinarm-standard-tests). To standardize interpretation, variables with negative scoring scales were inverted, so that, for all outcomes, a Z-score exceeding 1.65 reflects an impairment.

To examine the effects of time (T1, T2, T3) and lesion side (left vs. right hemisphere), linear mixed-effects models using restricted maximum likelihood (REML) were employed, with time, lesion side and time × lesion side as fixed effects and a random intercept for subjects to account for inter-individual variability. This approach allows the inclusion of all available data without listwise deletion, such that participants with incomplete observations were retained in the analysis. Missing data were handled under the assumption of missing at random (MAR). Sidak correction was used for post hoc comparisons. The normality of residuals from these models was assessed using Q-Q plots and the Shapiro–Wilk test. Results of task-specific variables that were identified as impaired in <10% of participants at T1 are presented in the Supplementary Materials (Tables S2 and S3).

All statistical analyses were performed using IBM SPSS Statistics version 30.0 (IBM Corp., Armonk, NY, USA), with an alpha level set at 0.05. Graphs were generated using GraphPad Prism version 10.6.1 (GraphPad Software, San Diego, CA, USA).

## 3. Results

### 3.1. Participants’ Characteristics and Impairments on Robotic Tasks

A total of 41 participants were assessed at T1. RVGR data from one participant were excluded at T1 due to a technical issue. Three participants were lost at T2, with RVGR data from one additional participant excluded. Six further participants were lost at T3. Reasons for dropout included voluntary withdrawal (*n* = 3), palliative care (*n* = 2), recurrent stroke (*n* = 1), distance constraint (*n* = 1), severe adhesive capsulitis of the shoulder (*n* = 1), and inability to contact (*n* = 1) (see Figure S1 in Supplementary Materials). Participants who dropped out differed significantly from those who completed the study only with respect to the lesion side, with a higher dropout rate observed among participants with a left-hemisphere lesion (see Table S1 in Supplementary Materials). 

The sample characteristics are summarized in Table 2. There was a higher proportion of neglect in participants with right-hemisphere lesions.

The proportions of occurrence of deficits for the VGR and RVGR tasks are presented in Table 3, for each measurement time and each variable.

At T1, only five participants (12%) were classified as impaired based on the VGR Task-score, and impairment rates for task-specific variables ranged from 2.4% to 22% participants, with the Reaction Time showing the highest proportion of deficits (22%). At T3, the proportion of deficits was 9.4% based on the Task-score. The Reaction Time remained the task variable with the highest proportion of deficits (18.8%).

In contrast, performance on the RVGR task was impaired in a large majority of participants at T1 (35, representing 87.5% of the sample). Task-specific variables with the highest proportion of participants showing impairments included Initial Direction Angle (77.5%), Correction Time (70%), Speed Maxima Count (67.5%), Movement Time (57.5%), and Path Length Ratio (57.5%). At T3, the Task-score remained impaired in 56.3% of the sample. The Initial Direction Angle (46.9%) and the Speed Maxima Count (37.5%) still showed the highest deficits among the task-specific variables, while much less frequent deficits were observed for the Correction Time (25%).

### 3.2. Effects of Time and Lesion Side on Performance on the Robotics Tasks

**Figure 2:**
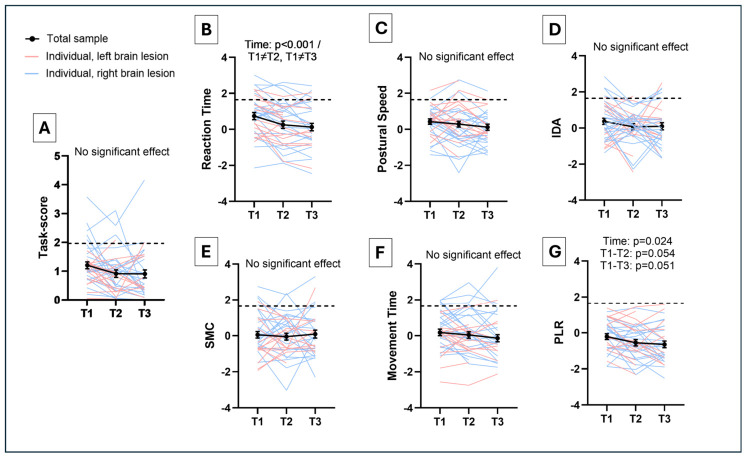
Performance across the three time points (T1, T2, T3) on the global Task-score (**A**) and task-specific variables (expressed as Z-scores, (**B**–**G**)) for the VGR task. Individual participant data are shown as red and blue curves for participants with left- and right-hemisphere lesions, respectively. The estimated marginal means and standard errors from the linear mixed models are shown as a black curve. IDA = Initial Direction Angle, SMC = Speed Maxima Count, PLR = Path Length Ratio. Values above the dashed line reflect impairment.

Estimated marginal means with 95% CI for the VGR task metrics are presented in Table S2 in the Supplementary Materials. They remained within normative ranges across all time points (Task-score < 1.96; Z-scores < 1.65; Figure 2 and Table S2 in Supplementary Materials). There were no statistically significant effects of time or lesion side on the VGR Task-score and on most task-specific variables. The two exceptions were Reaction Time and Path Length Ratio, for which a significant effect of time was observed (Figure 2).

**Figure 3:**
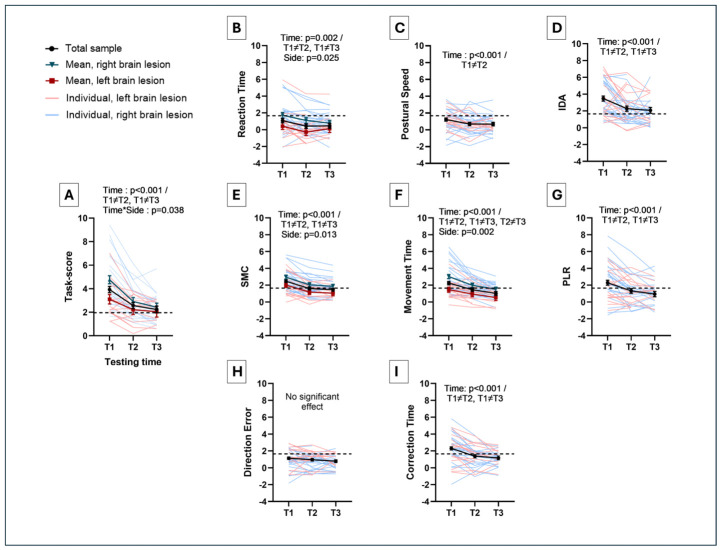
Performance across the three time points (T1, T2, T3) on the global Task-score (**A**) and task-specific variables (expressed as Z-scores, (**B**–**I**)), for the RVGR task. Individual participant data are shown as red and blue curves for participants with left- and right-hemisphere lesions, respectively. The estimated marginal means and standard errors from the linear mixed models are presented as black curves for the total sample. Red and blue curves represent the estimated marginal means for the subgroups of left- and right-hemisphere lesions, respectively, only when a significant effect of side or a side*time interaction was found (* denotes interaction). IDA = Initial Direction Angle, SMC = Speed Maxima Count, PLR = Path Length Ratio. Values above the dashed line reflect impairment.

In contrast, estimated marginal means for the RVGR task were outside normative ranges at T1 for the Task-score and 5/10 task-specific variables (Figure 3 and Table S3 in Supplementary Materials). Participants exhibited improvement (i.e., a significant effect of time) for the Task-score and all task-specific parameters except Direction Error and Min-Max Speed (Table S3 in Supplementary Materials). A significant time × lesion side interaction was observed for the RVGR Task-score: participants with right-hemisphere lesions performed worse at T1 but this difference decreased over time (Figure 3A). A significant effect of the lesion side (but no interaction) was observed for Reaction Time (Figure 3B), Speed Maxima Count (Figure 3E), and Movement Time (Figure 3F). For all these variables, participants with right-hemisphere lesions exhibited poorer performance compared to those with left-hemisphere lesions.

#### Sensitivity Analysis of Changes in RVGR Scores

Including both MoCA and visuospatial neglect as covariates (fixed effects) in the Task-score analysis preserved the time effect (*p* < 0.001), while the time × lesion side interaction was no longer significant (*p* = 0.07). When only MoCA was included, the interaction remained non-significant (*p* = 0.05), but the lesion-side effect became significant (*p* = 0.049). In contrast, including only neglect rendered both the interaction and lesion-side effects non-significant (*p* = 0.07 and *p* = 0.51, respectively).

For Reaction Time, including both covariates maintained a significant time effect (*p* = 0.003) but a non-significant lesion-side effect (*p* = 0.12). When added separately, results were similar (MoCA: *p* = 0.07; neglect: *p* = 0.05, for interaction effect).

For Speed Maxima Count and Movement Time, including both covariates did not alter the significant time (*p* < 0.001 for both) or lesion-side effect (*p* = 0.03 and *p* = 0.01, respectively).

## 4. Discussion

This study longitudinally compared the performance of the ipsilesional upper limb on two reaching tasks that were similar from a purely motor standpoint but differed in terms of cognitive–motor control: the VGR and RVGR. Participants generally performed well on the VGR task, whereas a high proportion exhibited impairments on the RVGR task, reflecting specific deficits in cognitive–motor control. Although performance on the RVGR task improved over time, impairments persisted at the 6-month follow-up in 56.3% of participants when considering the Task-score. This shows that full recovery of cognitive–motor control is not attained even at the chronic stage. Notably, participants with right-hemisphere lesions demonstrated greater deficits compared to those with left-hemisphere lesions, especially early after the stroke, highlighting the influence of lesion laterality on cognitive–motor control after stroke.

### 4.1. Contrasting Impairments on the VGR and RVGR Tasks

A high proportion of participants exhibited impairments on the RVGR task, with 87.5% classified as impaired based on the Task-score in the early subacute phase (T1), compared to only 12% on the VGR task. This highlights the specific challenges associated with cognitive–motor control. The continuous cognitive control required in the RVGR task substantially altered several components of motor control. For instance, high rates of deficits were observed in variables reflecting both feedforward (e.g., Initial Direction Angle) and feedback (e.g., Speed Maxima Count) components of movement. A study by Lowrey et al. using the same KINARM tasks also reported a higher occurrence of deficits for the RVGR than VGR in the ipsilesional upper limb of subacute stroke participants, and reported that deficits in both tasks were poorly correlated [5]. These findings support the hypothesis that deficits in cognitive–motor control are distinct and cannot be explained solely by underlying motor impairments.

When focusing on the two parameters that are specific to the RVGR task and capture cognitive–motor control, a higher proportion of participants showed deficits in Correction Time (70%) compared to Direction Errors (35%). These results, which are consistent with those of Lowrey et al. [5], suggest that while participants with subacute stroke were generally able to initiate movements in the appropriate direction, they required significantly more time to detect the errors or initiate the required motor corrections when errors occurred. This observation is interesting as this variable might help predict deficits in motor learning following stroke and response to motor-learning based intervention.

### 4.2. Longitudinal Performance and Lesion-Side Effects

In this study, participants’ performance on the VGR task remained stable over time, which is likely to be explained by the fact that very few participants demonstrated deficits at T1. These findings contrast with previous studies by Semrau et al., [28] Smith et al. [4] and Lowrey et al., [5] which reported substantially higher rates of occurrence of impairments (37%, 47% and 50%, respectively) and significant changes over time in VGR performance of the ipsilesional upper limb [4,5]. In the studies by Semrau et al. and Smith et al., assessments at T1 were performed at 1 week post-stroke [4,28]. However, Smith et al. observed changes only between 1 and 6 weeks post-stroke, with no significant differences at later assessments (12 and 26 weeks post-stroke) [4]. In the present study, the first assessment was conducted between 2 and 6 weeks post-stroke, which may have captured a period of relative stability and contributed to the low rate of occurrence of impairment (12%) observed. Similarly, Lowrey et al. conducted their first assessment between 1 and 10 weeks post-stroke [5]. Assessment at approximately 1 week post-stroke may have contributed to the higher rate of deficits observed in their study. Participants may also have experienced more severe strokes than those in our sample; however, the absence of assessment of severity limits comparisons. This higher proportion of deficits at T1 may explain the significant changes observed in their subgroup at 6 months [5].

Our results regarding the effect of lesion side on performance on the VGR task are consistent with previous studies, which also reported no effect [4,5,28]. This suggests that the presence of deficits in ipsilesional motor control is not strongly influenced by the side of the lesion.

For the RVGR task, participants demonstrated significant improvement over time; however, mean performance as reflected in the Task-score remained largely outside normative values (95% CI of healthy controls) at the follow-up around 29 weeks post-stroke. These findings are consistent with those of Lowrey et al., who observed persistent deficits in their smaller cohort [5], and extend previous studies reporting enduring deficits in similar cognitive–motor control tasks using computer- or tablet-based systems in individuals with chronic stroke [18,29].

Interestingly, participants with right-hemisphere lesions exhibited greater impairments compared to those with left-hemisphere lesions across multiple parameters, including Task-score, Reaction Time, Speed Maxima Count, and Movement Time, and these differences persisted several months post-stroke. This extends the findings of Lowrey et al., who reported similar lesion-side effects on the Task-score during the subacute phase (within 1 month post-stroke; [5]). Notably, this effect was not observed on the VGR task, suggesting that the right hemisphere may play a specific role in such cognitive–motor control involving a visuomotor task. These observations are in line with prior studies focusing on individuals with right-hemisphere damage using computerized assessments of similar tasks [18,29].

According to the hemispheric asymmetry model, the right hemisphere is proposed to play a primary role in regulating upper limb position and posture [30,31]. Under demanding visuomotor conditions, such as the RVGR task, participants with right-hemisphere lesions may have greater difficulty processing limb position to guide accurate movements, reflected in higher corrective movement demands as measured by Speed Maxima Count.

It is noteworthy that including MoCA scores and visuospatial neglect measures in the sensitivity analysis mitigates the effect of lesion side on RVGR performance. This suggests that one possible explanation for the observed lesion-side effect is the predominant role of the right hemisphere in visuospatial attentional control [22,23,24,32]. However, this interpretation cannot fully account for the lesion-side effect, as a significant influence of lesion side persists in some task-specific variables, such as Speed Maxima Count and Movement Time.

In a recent study, Rizvi et al. [33] demonstrated that transcranial excitatory direct current stimulation (tDCS) over the left primary motor cortex in healthy adults did not significantly affect RVGR performance, although it improved performance in a different cognitive–motor task (“Object Hit and Avoid”) requiring selective inhibition [34]. The authors attributed the lack of effect on the RVGR to the involvement of broader brain networks. Our findings suggest that these distributed networks may be more lateralized to the right hemisphere for the RVGR task, although neuroimaging studies are needed to confirm this hypothesis.

Overall, the observed effects of the side of lesion on the RVGR task provide further support for hemispheric asymmetry in cognitive–motor control, highlighting the specific contribution of the right hemisphere to visuomotor aspects of upper limb movement. Future studies, for example, using repetitive transcranial magnetic stimulation to produce lateralized “virtual lesions” in healthy volunteers, are warranted to better understand the neural mechanisms underlying these lateralized effects.

### 4.3. Limitations

First, the relatively small sample size in the subgroup of participants with left-hemisphere stroke may limit the generalizability of the findings.

Second, the study focused exclusively on a visuomotor cognitive–motor task. While the RVGR task provides valuable insight into visuomotor transformation and inhibitory control, inclusion of additional paradigms such as the “Object Hit and Avoid” task [34] could offer a more comprehensive understanding of lesion-side effects across different domains of cognitive–motor function. Furthermore, cognitive function and spatial neglect were evaluated solely at baseline (T1), without reassessment at follow-up time points (T2 and T3). Consequently, the trajectory of these deficits over time and their association with changes in cognitive–motor task performance remain unclear. Moreover, future studies should consider other behavioral confounders such as apraxia and affective status.

Third, stroke severity was not explicitly controlled for when comparing right- and left-hemisphere lesions. This may be relevant, as previous studies suggest that ipsilesional upper limb motor performance is associated with overall stroke severity [35]. Nevertheless, the lack of effect of lesion side on the VGR task, which primarily assesses motor function, suggests a degree of comparability in baseline motor performance of the ipsilesional upper limb between the two subgroups.

Lastly, some task-specific variables in both the VGR and RVGR tasks have been originally categorized to delineate feedforward and feedback mechanisms of motor control (see https://kinarm.com/kinarm-products/kinarm-standard-tests). However, we recognize that “Correction Time” in the RVGR task likely encompasses both feedforward and feedback components. As such, its interpretation as a purely feedforward measure should be treated with caution.

## 5. Conclusions

Stroke survivors exhibit significant impairments in cognitive–motor control of the ipsilesional upper limb, independent of pure motor deficits, with these impairments persisting into the chronic stage. Participants with right-hemisphere lesions demonstrated greater deficits than those with left-hemisphere lesions, suggesting a degree of hemispheric specialization in cognitive–motor control.

These findings underscore the importance of assessing cognitive–motor function in addition to motor performance alone, and highlight the need to integrate such measures into post-stroke rehabilitation. Future studies should investigate the neural substrates underlying performance on the RVGR task to better understand the observed hemispheric asymmetry. In addition, the potential predictive value of cognitive–motor control for motor learning and functional recovery after stroke warrants further exploration.

## Figures and Tables

**Table 1:** Description of the task-specific variables [see https://kinarm.com/kinarm-products/kinarm-standard-tests].

Categories	Variables	Definition
Postural Control	Posture Speed	Median hand speed when it should be at rest (median of all trials).
Visual Reaction	Reaction Time	Time interval between appearance of the target and movement onset (median of all trials).
First Movement (Feedforward)	Initial Direction Angle	Angular deviation between (a) a straight line from the hand position at movement onset to the one after the initial phase of movement and (b) a straight line from the hand position at movement onset to the target. The absolute value is calculated for each trial and the median of all trials is then used.
	Initial Distance Ratio	Ratio of (a) the distance the hand traveled during the initial phase of movement to (b) the one traveled between movement onset and offset (median of all trials).
	Direction Error *	Number of times the cursor initially moved away from the target.
	Correction Time *	When there is a direction error, the mean time before the initiation of the movement of the cursor towards the target. If the initial movement was toward the target, the value for that trial is zero. Otherwise, it is calculated as the amount of time from the target turning on to the time the cursor was the farthest from the target.
Corrective Movement (Feedback)	Speed Maxima Count	Number of hand speed maxima between movement onset and offset (mean of all trials).
	Min-Max Speed	Mean difference between all pairs of adjacent local hand speed minima and maxima between Max Speed and movement offset (mean of all trials).
Total Movement	Movement Time	Total time elapsed from movement onset to offset (median of all trials).
	Path Length Ratio	Ratio of (a) the distance traveled by the hand between movement onset and offset and (b) the straight-line distance between those hand positions (mean of all trials).

* These two parameters are specific to the Reverse Visually Guided Reaching task.

**Table 2:** Sample characteristics.

		Total Sample	Right-Hemisphere Lesion (*n* = 24)	Left-Hemisphere Lesion (*n* = 17)	*p*-Value *
Age, mean (SD)		64.59 (14.42)	66 (13.46)	62.59 (15.88)	0.46 ^a^
Sex, *n* (%)	Male	26 (63.4%)	16 (66.7%)	10 (58.8%)	0.75 ^b^
	Female	15 (36.6%)	8 (33.3%)	7 (41.2%)	
Handedness, *n* (%)	Left	3 (7.3%)	2 (8.3%)	1 (5.9%)	1.00 ^b^
	Right	38 (92.7%)	22 (91.7%)	16 (94.1%)	
Time since stroke (wks), mean (SD)		4.06 (1.15)	4.13 (1.27)	3.95 (0.98)	0.62 ^a^
Type of stroke, *n* (%)	Ischemic	31 (75.6%)	19 (79.2%)	12 (70.6%)	0.39 ^c^
	IPH	9 (22%)	4 (16.7%)	5 (29.4%)	
	Multiple	1 (2.4%)	1 (4.2%)	-	
Neglect (*n* = 38)	Yes	11 (26.8%)	10 (41.7%)	1 (5.9%)	0.012 ^b^
	No	27 (65.9%)	12 (50%)	15 (88.2%)	
MoCA	Median (IQR)	24 (20, 26)	24 (20, 26)	23.5 (17, 27)	0.92 ^d^
	Missing, *n* (%)	3 (7.3%)	2 (8.3%)	1 (5.9%)	

Note: wks = weeks, IPH = intraparenchymal hemorrhage, MoCA = Montreal Cognitive Assessment, IQR = interquartile range (1st, 3rd quartile). * Statistics between right- and left-hemisphere lesion groups, with independent *t*-test (a), Fisher’s exact test (b), chi-square test (c) and Mann–Whitney U test (d).

**Table 3:** Deficits in RVGR and VGR tasks.

Variables	RVGR			VGR		
	T1 (*n* = 40)	T2 (*n* = 37)	T3 (*n* = 32)	T1 (*n* = 41)	T2 (*n* = 38)	T3 (*n* = 32)
**Task-score**	**87.5%**	**64.9%**	**56.3%**	**12.2%**	**10.5%**	**9.4%**
Postural Speed	33.3% *	18.9%	15.6%	4.9%	10.5%	3.1%
Reaction Time	35%	16.2%	18.8%	22.0%	13.2%	18.8%
Initial Direction Angle	77.5%	48.6%	46.9%	12.2%	2.6%	12.5%
Initial Distance Ratio	5%	0%	0%	9.8%	7.9%	6.3%
Speed Maxima Count	67.5%	45.9%	37.5%	12.2%	5.3%	12.5%
Min-Max Speed	10%	13.5%	3.1%	2.4%	2.6%	6.3%
Movement Time	57.5%	43.2%	28.1%	12.2%	13.2%	9.4%
Path Length Ratio	57.5%	43.2%	28.1%	12.2%	13.2%	9.4%
Direction Error	35%	16.2%	18.8%			
Correction Time	70%	43.2%	25%			

* 1 participant data missing (*n* = 39). Values in bold (Task-scores) reflect overall task performance.

## Data Availability

The data presented in this study are available upon request from the corresponding author. The data are not publicly available due to restrictions related to ethical approval.

## Supplementary Materials

The following supporting information can be downloaded at https://www.mdpi.com/article/10.3390/brainsci16060595/s1. Table S1. Characteristics of dropout participants with comparison to the completers. Table S2. Results of the linear mixed models analysis of the performance on the Visually Guided Reaching task. Table S3. Results of the linear mixed models analysis of the performance on the Reverse Visually Guided Reaching task. Figure S1. Participants’ flow chart.

## Abbreviations

The following abbreviations are used in this manuscript:

VGR	Visually Guided Reaching
RVGR	Reverse Visually Guided Reaching
