# Supplementary material for: Deficits in Cognitive–Motor Control of the Ipsilesional Upper Limb in Subacute Stroke Assessed Using a Robotic Exoskeleton: A Longitudinal Study
Source: Brain Sci. 2026 May 30;16(6):595. doi: 10.3390/brainsci16060595 (PMC13297226; doi:10.3390/brainsci16060595)
Supplement: Supplementary file 1 [file brainsci-16-00595-s001.zip › brainsci-4283619-supplementary.pdf]

Supplementary material

Table S1. Characteristics of dropout participants with comparison to the completers

		Completers (n=32)	Non-completers (n=9)	Statistics
Age, mean(SD)		64.09(14.92)	66.33(13.16)	p=0.67[a]
Sex, n(%)	Male	20(62.5%)	6(66.7%)	p=1.00[b]
	Female	12(37.5%)	3(33.3%)	
Handedness, n(%)	Left	3(9.4%)	0	p=1.00[b]
	Right	29(90.6%)	9(100%)	
Less affected side, n(%)	Left	10(31.3%)	7(77.8%)	**0.02**[b]
	Right	22(68.8%)	2(22.2%)	
Time since stroke (wks), mean(SD)		3.97(1.24)	4.37(0.70)	p=0.37[a]
Type of stroke, n(%)	Ischemic	25(78.1%)	6(66.7%)	p=0.54[c]
	IPH	6(18.8%)	3(33.3%)	
	Multiple	1(3.1%)	0	
Neglect (n=38)	Yes	19(65.5%)	1(11.1%)	p=0.24[b]
	No	10(34.5%)	8(88.9%)	
MoCA, median(IQR)		24(20, 26)	20(16, 26)	p=0.18[d]

Note: wks = weeks, IPH = intraparenchymal hemorrhage, MoCA = Montreal Cognitive Assessment, IQR = interquartile range (1st, 3rd quartile).
* Statistical comparisons between groups, were performed with (a) independent t-test,(b) Fisher exact test, (c) chi-square test and(d) Man-Whitney U test.

Table S2. Results of the linear mixed models analysis of the performance on the Visually Guided Reaching task

	Estimated marginal means (95% CI)*			Time	Lesion side	Time×Lesion side
	T1	T2	T3			
Task-score	1.20 (0.96, 1.44)	0.91 (0.66, 1.16)	0.90 (0.63, 1.18)	p=0.06	p=0.24	p=0.26
Reaction time	0.74 (0.35, 1.13)	0.26 (-0.14, 0.66)	0.13 (-0.29, 0.55)	p<0.001, T1≠T2, T1≠T3	p=0.47	p=0.55
Postural speed	0.43 (0.13, 0.73)	0.29 (-0.02, 0.60)	0.12 (-0.22, 0.46)	p=0.27	p=0.10	p=0.28
Initial direction angle	0.37 (0.03, 0.70)	0.07 (-0.28, 0.42)	0.10 (-0.30, 0.49)	p=0.29	p=0.98	p=0.55
Initial distance ratio	0.29 (-0.14, 0.73)	0.21 (-0.24, 0.66)	0.09 (-0.41, 0.60)	p=0.76	p=0.62	p=0.45
Speed maxima count	0.05 (-0.32, 0.41)	-0.05 (-0.43, 0.33)	0.09 (-0.34, 0.51)	p=0.82	p=0.39	p=0.34
Min-max speed	-0.08 (-0.38, 0.22)	-0.33 (-0.64, -0.02)	-0.35 (-0.69, -0.00)	p=0.21	p=0.30	p=0.95
Movement time	0.18 (-0.18, 0.54)	0.04 (-0.33, 0.41)	-0.14 (-0.54, 0.25)	p=0.22	p=0.06	p=0.47
Path length ratio	-0.21 (-0.53, 0.11)	-0.55 (-0.88, -0.22)	-0.64 (-0.99, -0.28)	p=0.02$	p=0.67	p=0.94

Note: *Variables are expressed in Z-scores except Task-score and reported for total sample. $ = no significant effect in pairwise comparisons. CI = confidence interval

Table S3. Results of the linear mixed models analysis of the performance on the Reverse Visually Guided Reaching task

		Estimated marginal means (95% CI)*			Time	Lesion side	Time×Lesion side
		T1	T2	T3			
Task-score	Total sample	3.93 (3.41, 4.45)	2.57 (2.04, 3.09)	2.24 (1.67, 2.81)	p<0.001, T1≠T2, T1≠T3	p=0.06	p=0.038
	Right lesion	4.76 (4.09, 5.43)	2.91 (2.23, 3.58)	2.43 (1.75, 3.12)			
	Left lesion	3.1 (2.31, 3.89)	2.22 (1.41, 3.04)	2.05 (1.14, 2.96)			
Reaction Time	Total sample	1.07 (0.55, 1.59)	0.41 (-0.12, 0.94)	0.28 (-0.11, 1.01)	p=0.002, T1≠T2, T1≠T3	p=0.03	p=0.21
	Right lesion	1.72 (1.05, 2.40)	1.10 (0.43, 1.78)	0.78 (0.10, 1.46)			
	Left lesion	0.42 (-0.37, 1.21)	-0.28 (-1.09, 0.53)	0.12 (-0.77, 1.00)			
Postural speed	Total sample	1.20 (0.82, 1.57)	0.67 (0.29, 1.05)	0.66 (0.25, 1.08)	p=0.01, T1≠T2	p=0.95	p=0.44
	Right lesion	1.07 (0.58, 1.56)	0.77 (0.28, 1.26)	0.73 (0.24, 1.22)			
	Left lesion	1.33 (0.76, 1.89)	0.57 (-0.02, 1.16)	0.60 (-0.08, 1.28)			
Initial direction angle	Total sample	3.48 (2.89, 4.07)	2.28, 1.67, 2.89)	2.07 (1.39, 2.75)	p<0.001, T1≠T2, T1≠T3	p=0.33	p=0.77
	Right lesion	3.40 (2.63, 4.16)	1.99 (1.21, 2.76)	1.75 (0.96, 2.53)			
	Left lesion	3.55 (2.66, 4.45)	2.58 (1.63, 3.52)	2.40 (1.29, 3.50)			
Initial distance ratio	Total sample	1.15 (1.02, 1.27)	0.95 (0.82, 1.07)	0.85 (0.71, 0.99)	p=0.003, T1≠T2, T1≠T3	p=0.07	p=0.31
	Right lesion	-1.22 (-1.38, -1.06)	-1.08 (-1.24, -0.92)	-0.91 (-1.07, -0.75)			
	Left lesion	-1.07 (-1.25, -0.88)	-0.81 (-1.00, -0.62)	-0,79 (-1.02, -0.57)			
Speed maxima count	Total sample	2.47 (2.11, 2.84)	1.61 (1.24, 1.98)	1.46 (1.06, 1.85)	p<0.001, T1≠T2, T1≠T3	p=0.01	p=0.90
	Right lesion	2.95 (2.48, 3.43)	2.04 (1.57, 2.51)	1.86 (1.38, 2.34)			
	Left lesion	2.00(1.44, 2.55)	1.18 (0.61, 1.75)	1.05 (0.42, 1.68)			
Min-Max Speed	Total sample	0.39 (0.07, 0.72)	0.29 (-0.04, 0.63)	0.08 (-0.28, 0.45)	p=0.26	p=0.21	p=0.65
	Right lesion	0.30 (-0.12, 0.72)	0.07 (-0.35, 0.50)	-0.15 (-0.57, 0.28)			

	Left lesion	0.49 (-0.01, 0.98)	0.51 (0.01, 1.02)	0.31 (-0.27, 0.90)			
Movement time	Total sample	2.25 (1.85, 2.64)	1.47 (1.07, 1.87)	1.02 (0.59, 1.45)	$p<0.001$, T1≠T2≠T3,	$p=0.002$	$p=0.21$
	Right lesion	3.03 (2.52, 3.54)	1.99 (1.48, 2.50)	1.51 (0.99, 2.02)			
	Left lesion	1.46 (0.86, 2.06)	0.95 (0.33, 1.56)	0.53 (-0.16, 1.22)			
Path length ratio	Total sample	2.29 (1.72, 2.87)	1.32 (0.74, 1.90)	0.94 (0.32, 1.55)	$p<0.001$, T1≠T2, T1≠T3	$p=0.45$	$p=0.11$
	Right lesion	1.07 (0.58, 1.56)	0.77 (0.28, 1.26)	0.73 (0.24, 1.22)			
	Left lesion	1.33 (0.76, 1.89)	0.57 (-0.02, 1.16)	0.60 (-0.08, 1.28)			
Direction errors	Total sample	1.14 (0.81, 1.47)	0.96 (0.63, 1.30)	0.79 (0.42, 1.16)	$p=0.21$	$p=0.07$	$p=0.89$
	Right lesion	0.84 (0.41, 1.26)	0.73 (0.30, 1.15)	0.57 (0.14, 1.00)			
	Left lesion	1.44 (0.95, 1.94)	1.20 (0.69, 1.72)	1.02 (0.42, 1.62)			
Correction time	Total sample	2.30 (1.87, 2.73)	1.38 (0.94, 1.81)	1.18 (0.71, 1.65)	$p<0.001$, T1≠T2, T1≠T3	$p=0.96$	$p=0.41$
	Right lesion	2.43 (1.87, 2.99)	1.28 (0.72, 1.84)	1.12 (0.55, 1.68)			
	Left lesion	2.17 (1.52, 2.83)	1.47 (0.80, 2.15)	1.24 (0.48, 2.00)			

Note: *Variables are expressed in z-scores except task-score. CI = confidence interval.

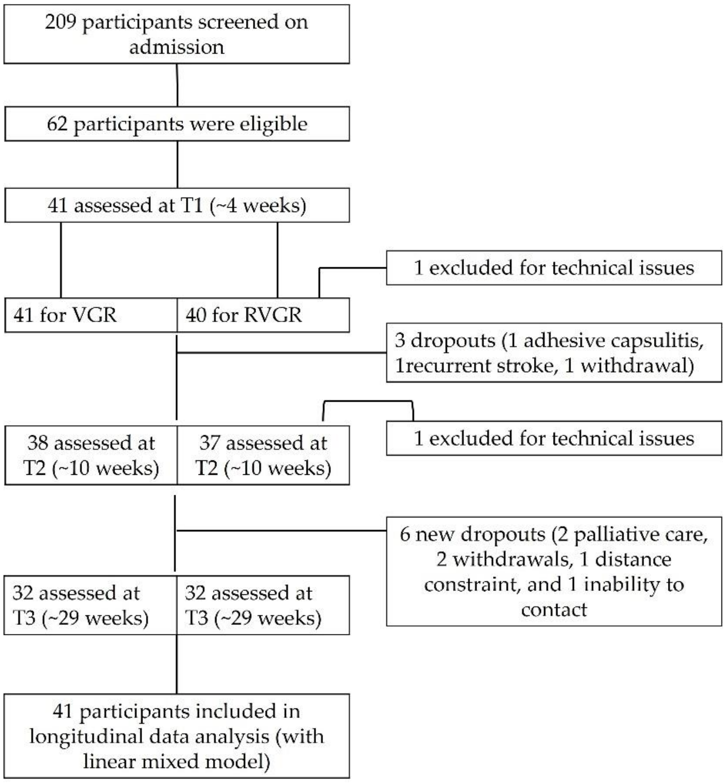

Figure S1. Participants' flow chart
